# Study designs and outcomes

**DOI:** 10.1007/s12471-026-02072-4

**Published:** 2026-08-10

**Authors:** Pim van der Harst

**Affiliations:** Department of Cardiology, University Medical Centre Groningen, Groningen, The Netherlands

This issue features two study protocols and two completed clinical investigations, each addressing important questions in cardiovascular medicine.

Ntantou et al. present the design of the CROWN study, using optical coherence tomography (OCT) to assess orbital atherectomy in severely calcified coronary lesions. The study will enroll 100 patients across six European centers. The primary endpoint, a minimum stent area of 5.5 mm^2^, correlates with post-procedural fractional flow reserve. Unlike ECLIPSE, which found no benefit from routine atherectomy, CROWN applies an OCT protocol in lesions unsuitable for balloon preparation alone [1].

Zwetsloot et al. describe the Dutch Cardiomyopathy Database, a new national registry coordinated by the Netherlands Heart Institute. It unites existing local cardiomyopathy registries under a shared data dictionary, starting with hypertrophic and dilated cardiomyopathy, and links national genetic data to support risk stratification and future patient identification [2].

Chatterjee et al. compare self-expanding and balloon-expandable valves in 56 patients undergoing transcatheter valve-in-valve procedures for a failing aortic bioprosthesis. Invasive gradients were similar between valve types, but echocardiography showed higher BEV gradients at discharge and 12 months, after valve-in-surgical-valve procedures [3].

Saouti et al. report the first 32 patients of a newly initiated Ross program, using a patient-tailored total root technique. Median age was 39 years, and 94% had a bicuspid valve. There was no operative or 30-day mortality; complications included one Bentall conversion and one urgent coronary intervention for button kinking. No patient had more than mild aortic regurgitation at discharge, supporting feasibility in a dedicated expert center with adequate case volume [4].

These studies underscore the importance of innovation, collaboration, and careful evaluation in improving cardiovascular care across the spectrum of disease.
